# Grey Matter Reshaping of Language-Related Regions Depends on Tumor Lateralization

**DOI:** 10.3390/cancers15153852

**Published:** 2023-07-28

**Authors:** Lucía Manso-Ortega, Laura De Frutos-Sagastuy, Sandra Gisbert-Muñoz, Noriko Salamon, Joe Qiao, Patricia Walshaw, Ileana Quiñones, Monika M. Połczyńska

**Affiliations:** 1 Neurobiology of Language Group, Basque Center on Cognition, Brain and Language (BCBL), 20009 Donostia-San Sebastián, Spain; l.defrutos@bcbl.eu (L.D.F.-S.); mariasandra.gisbert@esic.edu (S.G.-M.); 2 Department of Basque Language and Communication, University of the Basque Country, UPV/EHU, 48940 Bilbao, Spain; 3 Department of Radiology, University of California, Los Angeles, CA 92093, USA; nsalamon@mednet.ucla.edu (N.S.); jqiao@mednet.ucla.edu (J.Q.); 4 Department of Psychiatry and Biobehavioral Sciences, University of California, Los Angeles, CA 92093, USA; pwalshaw@mednet.ucla.edu (P.W.); mpolczynska@mednet.ucla.edu (M.M.P.); 5 IKERBASQUE, Basque Foundation for Science, Plaza Euskadi 5, 48009 Bilbao, Spain

**Keywords:** structural plasticity, VBM, brain tumor patients, language network

## Abstract

**Simple Summary:**

Brain tumors have a profound impact on the structural organization of the brain, particularly when located near language-related regions, resulting in impaired language processing. To investigate this phenomenon, we studied high-resolution MRI scans of patients with brain tumors in the left (dominant for language) and right (nondominant for language) hemispheres and compared them to controls. Specifically, we examined the grey matter volume in 10 language-related regions. Our findings demonstrate that brain tumors, regardless of their lateralization induce global volumetric changes in both the affected and contralesional hemispheres. These changes are influenced by the tumor’s lateralization and suggest that the brain undergoes structural reshaping to cope with the language deficits caused by the tumors. This study sheds light on the intricate relationship between brain tumors and language processing and contributes to the understanding of the mechanisms underlying neuroplasticity subsequent to lesion occurrence.

**Abstract:**

A brain tumor in the left hemisphere can decrease language laterality as assessed through fMRI. However, it remains unclear whether or not this decreased language laterality is associated with a structural reshaping of the grey matter, particularly within the language network. Here, we examine if the disruption of the language hubs exclusively affects the macrostructural properties of the contralateral homologues or whether it affects both hemispheres. This study uses voxel-based morphometry applied to high-resolution MR T1-weighted MPRAGE images from 31 adult patients’ left hemisphere, which is dominant for language. Eighteen patients had brain tumors in the left hemisphere, and thirteen had tumors in the right hemisphere. A cohort of 71 healthy individuals matched with respect to age and sex was used as a baseline. We defined 10 ROIs per hemisphere involved in language function. Two separate repeated-measure ANOVAs were conducted with the volume per region as the dependent variable. For the patients, tumor lateralization (right versus left) served as a between-subject factor. The current study demonstrated that the presence of a brain tumor generates global volumetric changes affecting the left language regions and their contralateral homologues. These changes are mediated by the lateralization of the lesion. Our findings suggest that functional mechanisms are supported by the rearrangement of the grey matter.

## 1. Introduction

The presence of a brain tumor can impair essential cognitive abilities, such as language. Consequently, the brain may reorganize to compensate for the presence of the lesion [1]. Patients with brain tumors can serve as an ideal pathological model to enhance our understanding of lesion-dependent plasticity. When the language hubs are damaged in this population, functional compensation involving the recruitment of the ipsilesional or contralesional regions has been observed to support recovery [2,3,4,5,6]. However, it remains poorly understood how the brain structurally responds to tumors harbored in the language hubs. While the scarce available evidence shows that patients with brain tumors in the language network have an increased grey matter (GM) volume in the contralateral regions [7,8,9], we do not yet know if structural changes are induced more globally, including ipsilaterally [10]. The goal of this work is to examine how tumor laterality affects language structures and their right-hemisphere homologues. To fulfill this goal, we examined the GM volume within the language network in the left hemisphere and its right homologues in patients with brain tumors affecting the language hubs. Furthermore, we determined whether changes in the macrostructural properties are evidenced through variations in the GM that could constitute a structural compensation mechanism.

Brain tumors located in the left language-dominant hemisphere can decrease language laterality as shown through functional magnetic resonance imaging (fMRI) assessments [6,7,8,9,10,11]. Decreased functional laterality has been linked to weaker activation during language tasks in the structures close to the lesion [12], increased contralesional activity in the right language homologues, or both [3,13,14,15]. In contrast, tumors located in the right hemisphere (which is not dominant for language) have been shown to have little to no effect on language activation during fMRI tasks [2,6,16]. It is yet to be determined whether functional compensation is accompanied by macrostructural reshaping within the language network.

At present, plasticity in patients with brain tumors remains fairly understudied. The resectability rates of the cortical and subcortical structures point to a far greater plastic potential of the GM than the white matter (WM) [17,18,19]. The GM volume can serve as an indicator of the structural alterations occurring in the brain. It is a sensitive measure and has been associated with functional consequences in various neurological conditions. Studying the fluctuations of the GM volume allows for a better understanding of the underpinnings of the functional changes. However, to our knowledge, only three studies have used voxel-based morphometry (VBM) with the GM volume as an index of structural plasticity in patients with brain tumors. In the first study, researchers investigated patients with gliomas affecting the left insula and the right insula [7]. Their findings revealed a significant increase in the volume of the contralesional insula in both groups, suggesting that the unaffected insula was likely recruited via an intact subcortical connectivity. Following a similar approach, [8] investigated the contralesional GM volumes associated with cognition in patients with temporal lobe gliomas. Compared with the healthy controls, the patients with left temporal tumors showed an increased GM volume in the right inferior temporal gyrus and right superior temporal pole, whereas the patients with right temporal tumors exhibited an increased GM volume in the left inferior temporal gyrus. More recently, structural alterations in the contralesional medial temporal lobe (MTL) were studied in patients with gliomas [9] using VBM. Patients showed decreased GM in this region. 

The current work examines the macrostructural properties of plasticity within the language network in patients with tumors affecting the left language-dominant hemisphere. For this purpose, the GM volume was analyzed as an index of structural plasticity. We measured the GM volume of previously identified language regions and their right homologues [20,21]. We hypothesized that patients with tumors in the left language-dominant hemisphere would show macrostructural changes in all the regions within the language network, including the right homotopic regions of interest and the close and far left ipsilateral regions. Changes in the GM volume would then be considered to be a sign of structural compensation to sustain one’s language ability after a brain lesion. We included two control groups: (1) patients with tumors in the right nondominant hemisphere in whom we predicted no structural alterations [2,6] and (2) 71 healthy volunteers matched with respect to age and sex. 

## 2. Materials and Methods

### 2.1. Participants

The clinical sample consisted of 31 patients (19 females, mean age = 47.6 years, SD = 13.9 years) who were diagnosed with intra-axial brain tumors. The sample presented in this study was accessed and retrospectively analyzed from a larger sample of patients published in [6]. Language impairment was assessed following a comprehensive approach that included both language production (including oral expression and writing) and comprehension (including auditory reception and reading). The evaluation of language performance was carried out by qualified technicians according to the standards of the language clinical fMRI assessment of the tumor program at the University of Los Angeles, California (UCLA). A summary of the patients’ characteristics can be found in Table 1, and detailed information for each patient can be found in Supplementary Materials (Table S1).

The data from all patients was obtained shortly before a planned surgery regardless of the existence of previous surgeries for some of the patients. Patients were divided into 2 groups: (1) left tumor group with 18 individuals (target group) and (2) right tumor group with 13 individuals (control group). This was based on medical records that could potentially affect language function. All patients were clinically classified as having left language dominance based on clinical conclusions from neurocognitive assessments, clinical language fMRI, and (in several cases) direct cortical stimulation. The recording of the pathological data was overseen by the Institutional Review Board at the University of California (code: K01DC016904), Los Angeles, following the Code of Ethics of the World Medical Association (Declaration of Helsinki) for experiments involving humans. 

In addition, healthy control data from 71 participants matched with respect to age and sex (43 women, mean age = 44.49, SD = 12.5) were used as a reference point. Specifically, we characterized the structural relationships among regions in the typical language network to interpret potential divergent patterns of structural reshaping in the patient groups. Healthy control participants were recruited, and their data were collected at the Basque Center on Cognition, Brain and Language (BCBL). The study protocol was conducted in accordance with the Declaration of Helsinki for experiments involving humans and was approved by the Ethics Board of the Euskadi Committee (code: 270220SM) and the Ethics and Scientific Committee of the Basque Center on Cognition, Brain and Language (BCBL). Informed consent was obtained from all participants involved in the study before the experiment.

### 2.2. Brief Overview of Data Acquisition and Analysis

**Figure 1:**
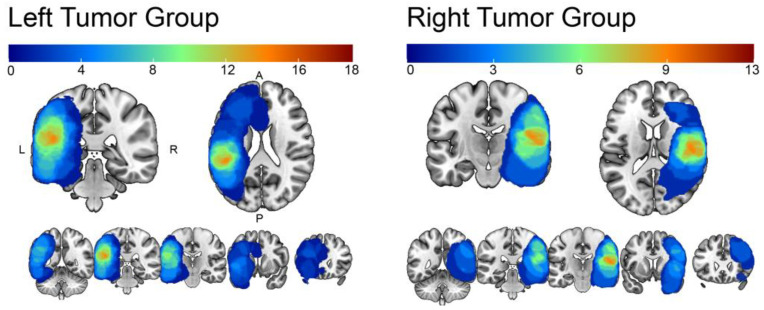
Lesion overlap per tumor group. The heatmaps—from red to blue—represent the number of overlapping tumors within the (**left**) and the (**right**) tumor groups in percentages. The maximum overlap is 14 out of 18 for the left group and 9 out of 13 for the right group.

**Figure 2:**
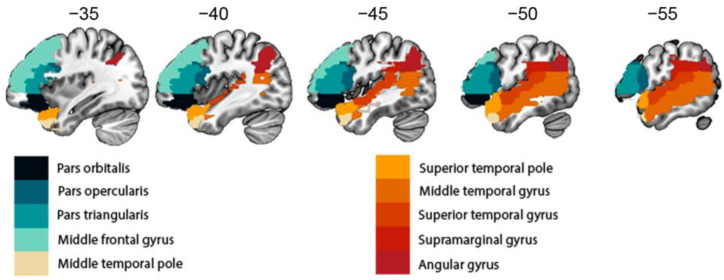
Picture created following AAL atlas parcellation with the selected language regions known to subserve language production (i.e., pars orbitalis, pars opercularis, pars triangularis, and middle frontal gyrus) and comprehension (middle temporal gyrus, middle part of the temporal pole, superior temporal gyrus, superior temporal lobe, supramarginal gyrus, and angular gyrus).

High-resolution MR images were obtained from patients and healthy controls using standardized parameters to minimize bias resulting from obtaining the images in different centers. Images were acquired using a 3T Siemens scanner with specific parameters for clinical data and healthy controls. Trained technicians manually delineated the lesions for the clinical population. The lesion overlap maps are displayed in Figure 1. For all participants, we used voxel-based morphometry (VBM) to quantify grey matter volume in brain regions involved in language production and comprehension (Figure 2). The estimation of grey matter volume in language-related regions in both patients with brain tumors and healthy participants allowed us to investigate the impact of a brain tumor on the neuroplastic mechanisms involved in language processing. A complete and detailed description of the acquisition and analysis process can be found in the subsequent sections.

MRI data acquisition. To minimize the possible bias caused by using images from different centers, the main acquisition parameters (e.g., magnet strength, model of scanner, and version of pulse sequence) were standardized. High-resolution MR T1-weighted MPRAGE images were obtained from the patients and controls using a 3T Siemens scanner (for clinical data: MAGNETOM Allegra with a 20-channel head coil) (for healthy controls: a MAGNETOM Prisma with a 64-channel head coil) with 176 slices of 1 mm isotropic resolution with matrix size of 256 × 256 (for clinical data: TR 1900 ms, TE 2200 ms, and flip angle of 9°) (for healthy controls: TR 2530 ms, TE 2360 ms, and flip angle of 120°). 

3D lesion reconstruction. Manual reconstruction of the lesion-affected area was conducted by two experts under the supervision of the main authors and taking as a reference the masks drawn by technicians at the University of Los Angeles, California. Masks were drawn slice by slice using the MRIcro-GL [22] free and open source software. To determine the affected area, we employed a combination of coregistered T1- and T2-weighted images using multiple intensity thresholds. The resulting binary mask derived from the manual reconstruction was used as a constraint to exclude the affected area from further statistical analysis. Furthermore, in order to avoid distortions produced by the normalization algorithm in pathological brains [23], the pipeline used for the current VBM analysis did not include normalization to MNI space. To determine GM in regions affected by the lesion and to estimate tumor volume (cm^3^) per participant, in-house MATLAB (2014b release, Mathworks, Inc., Natick, MA, USA) codes were developed using functions from Statistical Parametric Mapping (SPM12, Welcome Department of Cognitive Neurology, London, UK) and the related toolbox. Codes are available on github (see availability statement). Lesion overlap maps are displayed in Figure 1.

### 2.3. MRI Analyses

As mentioned, voxel-based morphometry (VBM) was performed using SPM12 in MATLAB for all participants. This semi-automatized neuroimaging method has been successfully used to quantify macrostructural brain changes—in volume or density—in longitudinal and cross-sectional studies on development and disease [24]. Previous results demonstrated that this type of multicenter study is methodologically feasible and reliable for the assessment of local changes in tissue integrity induced by a given pathological condition [25,26]. The processing pipeline was standardized for patients and healthy controls as described by [24]. 

For the healthy participants, a manual trimming of the images was performed. Then, the images were manually reoriented and shifted to set the anterior commissure, a bundle of white matter fibers that connect the anterior lobes of the brain, as the origin. Next, T1 MPRAGE-weighted images were segmented into the GM, WM, and cerebrospinal fluid (CSF) following the segmentation module in SPM12. The volumes of the native segmentations (GM, WM, and CSF) were computed and used to calculate the total intracranial volume (TIV) of each participant. For patients, we performed a subtraction operation using the binarized tumor masks to account for the presence of the lesion. To calculate the GM volume per region, we used the automated anatomical labeling (AAL) atlas [27]. The volume of each of the 116 ROIs of the AAL atlas was calculated and divided by the TIV. The use of proportionally scaled scores (volume per ROI divided by TIV) instead of using GM segmentations minimized potential bias due to variables that we were not able to control for and could potentially affect each individual differentially. 

To investigate the impact of brain tumors on neuroplastic structural mechanisms affecting ipsilateral and contralateral language areas, 10 regions that are critically involved in language processing were selected [20]. We included pars orbitalis, pars opercularis, and pars triangularis within the inferior frontal gyrus (IFG), middle frontal gyrus, middle temporal gyrus (MTG), middle temporal pole, superior temporal gyrus (STG), superior temporal pole, supramarginal gyrus (SM), and angular gyrus (Figure 2). 

### 2.4. Statistical Approach

Statistical comparisons were performed, keeping patients and healthy controls in separate designs in order to avoid potential effects due to the differences between the scanners inherent to the different populations. A repeated measures ANOVA was performed with GM volume for the 10 ROIs weighed by the TIV (for patients: mean TIV = 1536.08, SD = 237.36) (for healthy controls: mean TIV = 1419.92, SD = 158.22) as dependent variable. Two within-subject factors were included: (1) ROI lateralization (right/left) and (2) regions of interest (ROIs) (10 ROIs were considered, including frontal, temporal, and parietal areas). As patients had lesions affecting either the left or the right hemisphere, lesion lateralization was considered as a between-subject factor controlling for lesion volume and tumor grade. We also included for both groups—patients and healthy controls—age and sex as nuisance covariates. By including age and sex as nuisance covariates, we controlled for any variance in the data that could be attributed to sex- and age-related factors. Pairwise comparisons were calculated as a post hoc analysis, applying Bonferroni correction for multiple comparisons. We observed that ROI volumes appeared to be unaffected by the presence of language impairment. This is visually demonstrated in the graphical representation of region-specific volume distributions of patients with and without language impairment and is available in the Supplementary Materials (Figure S1).

## 3. Results

### Structural Reshaping in Patients with Brain Tumors

**Figure 3:**
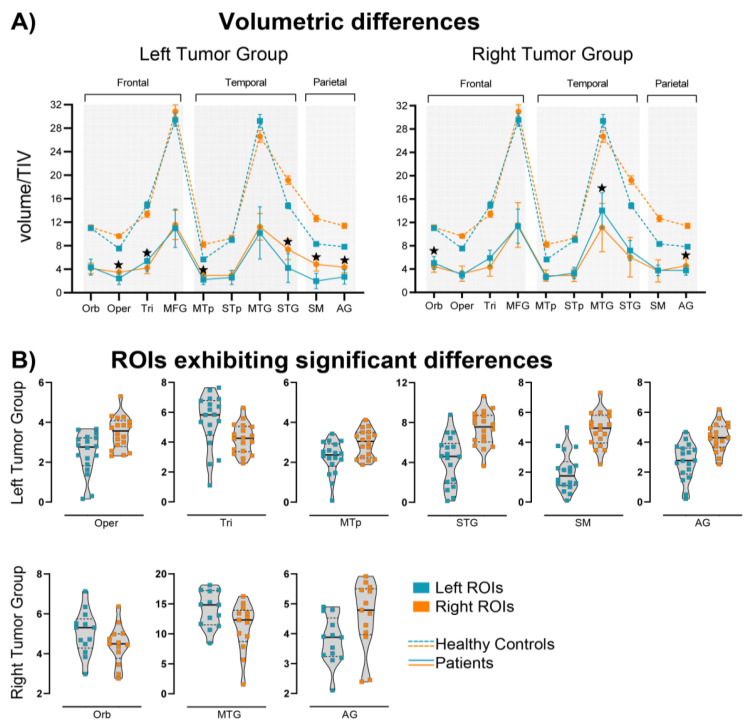
Volumetric differences. (**A**) Line charts show volumetric differences for each group of patients considering the 10 ROIs (**left** vs. **right**). The ratio between volume per region and TIV values is also shown for healthy controls as indicated by dotted lines. Stars indicate that the comparison of contralateral regions reached significance after Bonferroni correction (*p* < 0.002). (**B**) Violins represent regions showing a significant difference with squares representing each patient. Abbreviations stand for pars opercularis (Oper), pars orbitalis (Orb), pars triangularis (Tri), middle temporal pole (MTp), superior temporal gyrus (STG), middle temporal gyrus (MTG), supramarginal gyrus (SM), and angular gyrus (AG).

After performing Greenhouse–Geisser sphericity correction, we found double and triple interactions (illustrated in Figure 3). We found that the patients with tumors in the left hemisphere had greater volume in the right ROIs (contralesional) relative to the same ROIs in the left hemisphere (ipsilesional) as shown by the double interaction between ROI lateralization (right/left) and lesion lateralization (right/left) (F(1) = 25.93, *p* < 0.001). The triple interaction of ROI lateralization × ROIs × lesion lateralization (F(9) = 6.29, *p* < 0.001) was also significant. A table with the complete results of the ANOVA can be found in the Supplementary Materials (Table S2). Pairwise comparisons were calculated in a post hoc analysis (Table 2). For the patients in the left tumor group, after applying Bonferroni correction, 6 out of 10 structures exhibited larger volumes in the right hemisphere relative to the left: the supramarginal gyrus, angular gyrus, superior temporal gyrus, middle temporal pole, and pars opercularis within the IFG. One ROI displayed a reversed result with greater volume on the left than the right side (the pars triangularis within the IFG) (Figure 3B). The individuals with tumors in the right hemisphere had similar volume values for the left and right ROIs, with three larger structures on the left (contralesional) (see Figure 3B). These areas were the middle temporal gyrus, pars orbitalis, and pars triangularis. The angular gyrus was the only structure that had greater volume in the right hemisphere compared to the left. 

Both groups of patients and the healthy controls showed similar relations of the volumetric differences between the left and right ROIs as can be seen in Figure 3. The pars triangularis exhibited the same pattern for all the patients and healthy controls: greater volume in the left hemisphere. The middle temporal gyrus was bigger in the left hemisphere for the right tumor group and healthy controls but not for the left tumor group. The remaining regions were all bigger in the right hemisphere for all the participants, except for the pars orbitalis, which was bigger in the left hemisphere for the right tumor group. The healthy participants showed a complete asymmetrical lateralization pattern, whereas the brain tumor patients did not present asymmetries for some of the regions. For the left tumor group, 4 out of the 10 selected regions were different when compared to the control group: the pars orbitalis, middle frontal gyrus, superior temporal pole, and middle temporal gyrus. In the case of the right tumor group, seven regions were different from those of the healthy controls: the pars orbitalis (bigger in the left instead of the right), pars opercularis, middle frontal gyrus, middle temporal pole, superior temporal pole, superior temporal gyrus, and supramarginal gyrus.

## 4. Discussion

In the present VBM study, we investigated the structural flexibility of the language network in patients with brain tumors affecting the language-dominant hemisphere. To identify the structural changes dependent on tumor laterality, we analyzed two groups of patients: patients with left tumors and patients with right tumors. Furthermore, a group of healthy controls was included to provide the standard lateralization pattern of the language network to enable the interpretation of the potential compensatory effects triggered by the growth of a brain lesion. Previous studies only analyzed the structural alterations in the homologues brain sites that were contralesional to the tumor: one focused on the insula [7], and two focused on the temporal lobe [8,9]. These regions are known to be implicated in several cognitive processes rather than being specific for language [20,21]. Conversely, one of the strengths of this research is that it also accounts for the ipsilateral regions in the left hemisphere, either those adjacent to the tumor or the ipsilateral regions that are more distant from the lesion.

Three main findings can be highlighted, which we summarize here and discuss in more detail below. First, all of the patients, regardless of tumor laterality, showed a global change in the left language-dominant network and its contralateral counterpart compared to the control group. Second, contrary to what had been expected, a brain tumor induced neuroplastic mechanisms in both patient groups (left and right tumors), not just in the patients with tumors in the left language-dominant hemisphere. Third, both tumor groups displayed different patterns of regional structural lateralization. Overall, these findings suggest that the growth of a brain tumor induces neuroplastic mechanisms in the language regions that are not limited to patients with tumors in the left language-dominant hemisphere, with some specificities depending on tumor lateralization.

Our first main finding was that there were GM dissimilarities between the patients and healthy controls that were most likely induced by the presence of the tumor. This result is consistent with the previous findings focused on the structural changes of the contralesional language counterpart [7,8,9]. Furthermore, our results demonstrate that structural reshaping is not circumscribed to areas contralesional to the left language network, but instead, it spreads across both hemispheres. The entire network seems to change to cope with damage and to maintain its functions. In spite of the limitations of the VBM method to explain the physiology underlying neuroplastic mechanisms, a glioma can be considered to cause metabolic stress. Therefore, it is plausible to hypothesize that its removal might induce neuronal plasticity. As has been suggested before, this process is probably accompanied by secondary synaptogenesis and dendritic sprouting, which might affect all the regions within a network [28]. Likewise, cerebrovascular reactivity (CVR) could also be affected. In fact, impaired CVR has been found within the lesion and in the whole brain for patients with diffuse gliomas [29].

Second, although structural changes in the cortex were expected only in the left tumor group, both tumor groups showed a similar structural pattern in which the left and right ROIs were affected regardless of the tumor location. Therefore, our results concur with recent studies that propose a shift from the traditional view of language being strongly left-lateralized with the right hemisphere merely supporting it [30]. Instead, the right hemisphere has been demonstrated to be actively involved in the language functions, including second language learning [31,32] and language recovery after a brain lesion [33,34,35]. As neuroplasticity seems to affect the language network (including the right nondominant hemisphere), additional attention should be given to the right hemisphere in the assessment of language from a clinical standpoint. This idea challenges the current clinical standards in which intraoperative brain mapping in patients with brain tumors in the right hemisphere only accounts for the social, somatosensory, and visuospatial processes [36,37,38,39]. Ignoring the right hemisphere’s contributions to language increases the risk of language deficits after surgery as reported in previous studies [40]. For future research and based on our findings, we predict a better recovery prognosis for those patients whose structural lateralization patterns within each region resemble those of the healthy controls. Ultimately, furthering our understanding of the global macrostructural reshaping of the language network in patients with brain tumors relative to healthy controls may help target future approaches to language therapy. 

Our third main finding was that there were commonalities and differences in the structural relationships between the homologous regions for both tumor groups in contrast to the healthy controls. With respect to the lateralization pattern in the healthy controls, the right structures showed greater volume than the left counterparts, with the exception of the pars triangularis within the IFG and the middle temporal gyrus. The leftward asymmetry of these two critical language hubs has been well documented in postmortem and in vivo specimens [41,42,43]. In a study investigating the arcuate fasciculus (AF) specifically, they observed that patients with brain tumors who showed a symmetric or right-lateralized AF presented no language deficits, suggesting that the structure of the language right homologues can have an impact on recovery [44]. Nonetheless, the rightward or leftward asymmetry pattern of each of the regions is still under debate and is actively being studied [45,46]. Asymmetries are a core element of the typical organization of the brain. Cortical symmetries have been linked to neuropsychiatric and cognitive disorders [47,48]. In addition, to evaluate the relationship between the structural asymmetry of the middle temporal gyrus and functional language laterality, Reynolds et al. (2019) [49] investigated the structural and functional development of language asymmetry in 117 healthy children across early childhood. They demonstrated that the macrostructural asymmetry of the arcuate fasciculus—the WM tract connecting the IFG and the middle temporal gyrus—is pronounced from the age of 2 years and increases even more over time [49]. Likewise, language models propose the IFG to be left lateralized, whereas other regions are more bilateral [20]. In our study, the healthy participants showed asymmetries for all the regions, whereas the patients did not. However, the language hubs considered crucial for maintaining the network topology, such as the pars triangularis within the IFG and the angular gyrus [17], exhibited the same pattern in all the patients and healthy controls, demonstrating the capacity of the brain to change to accommodate language functions. This work demonstrates that, in addition to the previously documented functional reshaping in individuals with brain tumors when the language hubs are damaged [3,5,6], structural changes also occur in both patients with tumors in the left (language-dominant) hemisphere and in patients with tumors in the right hemisphere. Furthermore, we demonstrated these changes with GM volume indexes, as GM has been stated to have a great plastic potential [17,18]. These changes are likely representative of the compensatory mechanisms. On a similar note, structural changes should also be studied in different populations, such as stroke patients, as previous functional evidence has shown that the location of the lesion determines the differential involvement of the ipsilesional or contralesional areas [50].

Limitations and future directions. Three limitations of the present work should be mentioned, and we are already working towards addressing them in future work. First, our sample size was limited by our access to data from this specific population, which constrains the amount of variability among the patients. We acknowledge the heterogeneity of our patient sample, yet these types of samples capture the diversity and complexity of the patient population encountered in clinical practice. This enhances the generalizability of our findings and provides insights into the structural alterations associated with various brain tumors. However, to mitigate the potential effects of confounding variables, we included sex, age, tumor type, and tumor size as nuisance covariates. However, they may have independent effects which cannot be disambiguated from those related to the sample size. To solve this, larger cohorts are needed to replicate the findings, increase the statistical power and sensitivity, detect group differences, and investigate possible relations with interindividual variables, perhaps via large-scale multicenter collaborations. Larger samples would mean having access to more homogeneous populations that would allow us to draw stronger conclusions. It would also aid in the development of guidelines for the presurgical assessment of patients with brain tumors affecting not only the left language-dominant hemisphere but also its right counterpart. 

The second limitation is the cross-sectional nature of the current study, which does not allow for examining how neuroplastic mechanisms unfold over time in individuals with tumors. Additionally, in this study, we focused on assessing the participants at a single timepoint; therefore, we do not provide information about recovery and could not state that the structural alterations are a compensatory mechanism. Nonetheless, cross-sectional studies provide useful contributions, and in this case, one of the strengths of our study lies in having the healthy sample to discuss in relation to the patients’ data. This led us to detect the global changes most likely caused by the tumor growth over time. However, longitudinal studies are also needed. Such studies would result in direct and reliable evidence on how structural neuroplastic mechanisms occur in each individual as well as provide detailed measures of their cognitive performance throughout the process. This would help delineate the neuroplasticity mechanisms for recovery and facilitate the identification of neuroimaging predictors for postoperative prognosis. The use of connectivity measures in future longitudinal studies is fundamental to disentangle (1) whether only the regions that are interconnected structurally and/or functionally follow the same neuroplastic patterns overtime and (2) if those patterns respond to fluctuations in the entropy of the system as suggested by the global difference encountered in our study.

The third limitation was that the patients and healthy participants were collected from different centers, which prevented us from making a formal comparison. In the future, it would be desirable to test both patients and healthy participants from the same center to avoid any potential confounds.

## 5. Conclusions

To our knowledge, this research is the first to show that a brain tumor affecting the left language network or its right homologue induces global structural reshaping, highlighting the brain’s plasticity. Our work emphasizes the need to extend the scope of presurgical and intraoperative brain mapping in patients with tumors since the impact of a brain lesion appears to be more global. Intraoperative mapping should be designed to respect the anatomical substrate that is already going through neuroplastic processes to promote recovery and ultimately minimize the long-term deficits. Future multicenter longitudinal studies regarding the impact of a brain tumor on the neuroanatomy of language are needed to broaden our understanding of the processes of structural and functional compensation in individuals with brain tumors.

## Figures and Tables

**Table 1:** Patients’ characteristics.

	Left Tumors (*n* = 18)	Right Tumors (*n* = 13)
**Handedness**		
Right	16	4
Left	1	7
Ambidextrous	1	2
**Tumor type WHO**		
Anaplastic astrocytoma	3	2
Glioblastoma multiforme	6	5
Metastatic	2	0
Oligoastrocytoma	3	1
Oligodendroglioma	3	2
No data	1	3
**Tumor grade**		
Low grade	6	7
High grade	11	4
No data	1	2
**Previous surgery**		
Yes	4	3
No	12	9
No data	2	1
**Language impairment**		
Yes	12	8
No	4	5
No data	2	0

**Table 2:** Paired sample *t*-test for each group (R > L).

Region	Left Tumor Group			Right Tumor Group			Healthy Controls		
	T	*p*	Cohen’s d	T	*p*	Cohen’s d	T	*p*	Cohen’s d
**Pars orbitalis**	−1.11	0.282	−0.26	−4.50	<0.001 *	−1.25	3.50	<0.001 *	0.42
**Pars opercularis**	6.29	<0.001 *	1.48	0.55	0.590	0.15	55.24	<0.001 *	6.56
**Pars triangularis**	−4.33	0.001 *	−1.02	−3.65	<0.003 *	−1.01	−36.11	<0.001 *	−4.29
**Mid frontal gyrus**	1.55	0.139	0.37	0.22	0.827	0.06	21.84	<0.001 *	2.59
**Mid temporal pole**	4.13	<0.001 *	0.97	0.93	0.369	0.26	77.36	<0.001 *	9.18
**Sup temporal pole**	1.88	0.078	0.44	−1.46	0.171	−0.40	10.45	<0.001 *	1.25
**MTG**	1.13	0.275	0.27	−4.67	<0.001 *	−1.30	−40.38	<0.001 *	−4.79
**STG**	5.40	<0.001 *	1.27	−1.66	0.123	−0.46	76.76	<0.001 *	9.11
**SM**	6.51	<0.001 *	1.53	−0.25	0.811	0.07	90.24	<0.001 *	10.71
**Angular gyrus**	4.85	<0.001 *	1.14	4.89	<0.001 *	1.36	92.25	<0.001 *	10.95

Asterisks represent significant comparisons after Bonferroni correction. Effect size is given by standardized Cohen’s d.

## Data Availability

The data presented in this study are available on request from the corresponding author. The data are not publicly available due to the data-sharing policies of the different institutions involved concerning vulnerable clinical information. Codes are available on GitHub: https://github.com/lmansoo (accessed on 17 July 2023).

## Supplementary Materials

The following supporting information can be downloaded at https://www.mdpi.com/article/10.3390/cancers15153852/s1, Figure S1: ROI volume distribution and presence of language impairment; Table S1: Patients’ detailed demographic information; Table S2: Complete repeated measures ANOVA for the patient’s group.
