# Supplementary material for: Grey Matter Reshaping of Language-Related Regions Depends on Tumor Lateralization
Source: Cancers (Basel). 2023 Jul 28;15(15):3852. doi: 10.3390/cancers15153852 (PMC10417790; doi:10.3390/cancers15153852)
Supplement: Supplementary file 1 [file cancers-15-03852-s001.zip › cancers-2474800-supplementary.pdf]

Table S1. Patient’s detailed demographic information.

P	Age	Handedness	Sex	Lesion location	Tumor Grade	Previous Surgery	Seizures	Language impairment	Level of impairment	Tumor type WHO
P1	22	Right	Female	Left	Low	No	No	Yes	Mild	Anaplastic astrocytoma
P2	55	Right	Female	Left	High	No	Yes	Yes	Moderate	Anaplastic astrocytoma
P3	61	Ambidextrous	Female	Left	High	Yes	Yes	Yes	Mild	Anaplastic astrocytoma
P4	41	Right	Female	Left	High	No	Yes	No	No	Glioblastoma
P5	52	Right	Female	Left	High	No	Yes	Yes	Moderate	Glioblastoma
P6	36	Right	Female	Left	High	No	No	Yes	Moderate	Glioblastoma
P7	43	Right	Female	Left	High	Yes	Yes	Yes	Moderate	Glioblastoma
P8	48	Right	Female	Left	High	No	Yes	Yes	Mild	Metastatic
P9	53	Left	Female	Left	-	-	Yes	-	-	-
P10	51	Right	Female	Left	Low	No	Yes	Yes	Mild	Oligoastrocytoma
P11	59	Right	Female	Left	Low	Yes	Yes	No	No	Oligodendroglioma
P12	53	Right	Female	Left	High	No	Yes	Yes	Mild	Oligodendroglioma
P13	62	Right	Male	Left	High	No	No	Yes	Mild	Glioblastoma
P14	26	Right	Male	Left	High	No	Yes	Yes	Mild	Glioblastoma
P15	75	Right	Male	Left	Low	No	No	Yes	Severe	Metastatic
P16	46	Right	Male	Left	Low	No	No	No	No	Oligoastrocytoma
P17	25	Right	Male	Left	Low	-	Yes	-	-	Oligoastrocytoma
P18	40	Right	Male	Left	High	Yes	-	No	No	Oligodendroglioma
P19	52	Left	Female	Right	Low	No	No	No	No	Anaplastic astrocytoma
P20	58	Left	Female	Right	Low	No	-	Yes	Moderate	Anaplastic astrocytoma
P21	57	Ambidextrous	Female	Right	Low	No	Yes	Yes	Mild	Glioblastoma
P22	64	Left	Female	Right	High	No	No	No	No	Glioblastoma
P23	31	Right	Female	Right	High	Yes	-	Yes	Mild	Glioblastoma
P24	48	Right	Female	Right	-	-	-	Yes	Mild	-
P25	54	Left	Female	Right	Low	No	Yes	Yes	Moderate	Oligodendroglioma
P26	67	Ambidextrous	Male	Right	High	Yes	No	No	No	Glioblastoma
P27	28	Right	Male	Right	High	No	Yes	Yes	Mild	Glioblastoma
P28	22	Right	Male	Right	-	No	Yes	Yes	No	-
P29	44	Left	Male	Right	Low	Yes	-	No	No	-
P30	65	Left	Male	Right	Low	No	Yes	No	No	Oligoastrocytoma
P31	44	Left	Male	Right	Low	No	Yes	Yes	Mild	Oligodendroglioma

Table S2. Repeated measures ANOVA for the patient sample.

	Sum of Squares	df	Mean Square	F	p	η²
ROI lateralization	2.099	1	2.099	1.354	255	2.30E-04
ROI lateralization x Lesion lateralization	11.052	1	11.052	7.126	0.013*	1
ROI lateralization x Sex	0.04	1	0.04	26	874	4.35E-06
ROI lateralization x Age	298	1	298	192	664	3.27E-05
Residuals	41.872	27	1.551			
ROIs	3.764.081	1.214	3.100.526	87.726	< .001*	412
ROIs x Lesion lateralization	247.389	1.214	203.778	5.766	0.017*	27
ROIs x Sex	20.393	1.214	16.798	475	532	2
ROIs x Age	9.108	1.214	7.502	212	695	9.97E-04
Residuals	1.158.501	32.778	35.343			
ROI lateralization x ROIs	112.841	2.986	37.793	25.295	< .001*	12
ROI lateralization x ROIs x Lesion lateralization	27.241	2.986	9.123	6.106	< .001*	3
ROI lateralization x ROIs x Sex	4.471	2.986	1.497	1.002	396	4.89E-04
ROI lateralization x ROIs x Age	2.435	2.986	816	546	652	2.67E-04
Residuals	120.447	80.617	1.494			

Results are corrected by Greenhouse-Geisser sphericity correction. Asterisks depict significant results

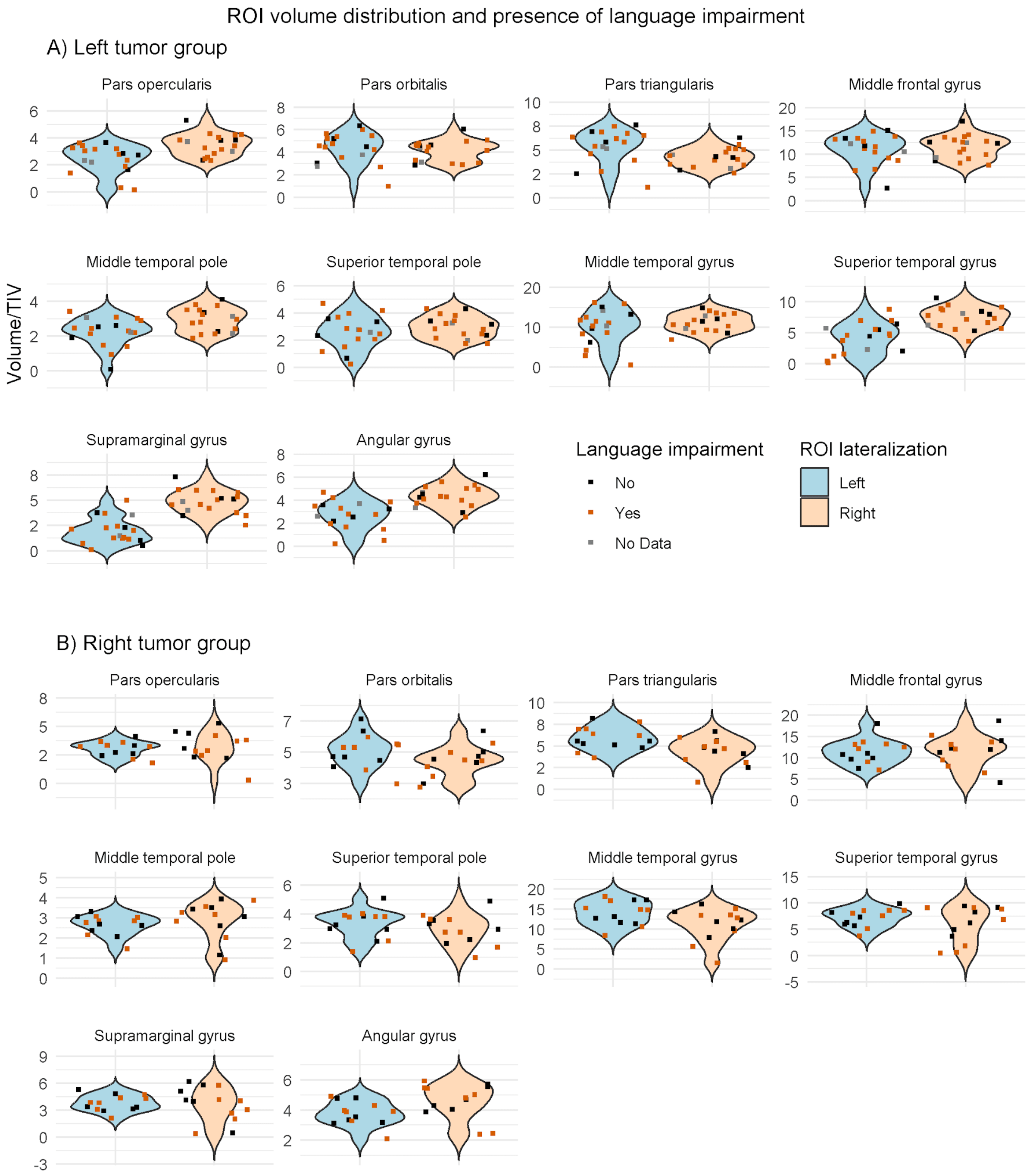

Figure S1. ROI volume distribution and presence of language impairment. Violins represent region-specific volume distribution for the 10 ROIS in both left and right hemispheres for each group of patients (sections A and B). The values are shown weighed by the TIV to account for individual variability. Each patient is denoted by a square, color-coded to indicate the presence or absence of language impairment. Missing data points are represented by gray squares.
